# The Structure and Functions of the Female Breast as They Relate to Its Health, Derangement and Disease

**Published:** 1846-07

**Authors:** 


### The Structure and Functions of the Female Breast as they relate to its Health, Derangement and Disease. By *E. W. Tuson*, F.R.S. F.L.S. Surgeon to the Middlesex Hospital. London: John Churchill, 1846.

We have always thought it very desirable that surgeons or medical officers to the larger hospitals in this country should publish the result of their experience, when they have filled their stations for fifteen or twenty years.

We conceive that few things would be of greater practical benefit to the profession than a more general acquiescence in this plan. Mr. Tuson, since the year 1823, has been attached to the Middlesex Hospital, and it cannot be denied that he must have had great opportunities for the pursuit of practical surgery. His treatise is on a very interesting and extensive subject, which has already been well written on by the best surgeons of the present and a former day. But while we are willing to accord to Mr. Tuson praiseworthy motives in undertaking the task in question, we regret to add our conviction that his monograph is a very ordinary production. He has managed to work it out into a good sized volume numbering 485 pages, and we are quite convinced that it might well have been condensed into a fourth part of the size. It has rarely fallen to our lot to read a more confused and clumsily written Preface than that which is prefixed to this volume. If the author really had a well-defined meaning in one half of it, the language he has used has been a most effectual cloak to conceal it. We take at random the following quotation.

"The opportunities since the period I entered the Hospital, in the year 1823, have been very numerous, both of watching this disease (cancer) and observing the effect of many remedies that have been recommended; still I must confess that I feel little satisfaction with the progress made in such a number of years; but, urged on of late by the hope of alleviating nature's sufferings, I have been more than particularly active in my researches, and feel a greater gratification in having accomplished much more in a shorter space of time. Promises are easily made, when unaccompanied by diligence and perseverance; but, with a full determination to enter into the subject in question, let us hope that we may realize more advantages in less time by following up the course already in progress, and by making use of what has already been acquired." P. ix.

The first Chapter of the Book is occupied with an Anatomical Examination into the Structure and Functions of the Mammary Glands, and the Diseases of the Breast are arranged under three classes. Class I. includes the functional and painful affections of the Breast. Class II. comprises those organic lesions of the Breast generally occurring independently of Inflammation. Class III. includes another variety of organic lesion—viz. Tumours or Formations of a Malignant and Contaminating Nature. A number of Cases illustrating the use of certain remedies in the Cure of Cancer in different parts of the body concludes the book.

After a brief description of the anatomy of the mammary glands, our author "takes a short survey of the changes these glands undergo during certain periods of life, such as puberty, menstruation, parturition, (gestation ?) lactation and the decline of life." When speaking of the properties of human milk, Mr. Tuson digresses into a consideration of the value of Proteine as a medicine.

"Five pounds of the fibre of beef will produce about three ounces of proteine. Its quality is highly nourishing, and it may be given in sugar : to weakly infants it bids fair to be of the greatest service, and in delicate constitutions, when the blood is insufficient for the secretion of a healthy milk, there is little doubt that it will be highly beneficial, as it is identical in composition with the chief constituents of blood, animal fibrine, and albumen, and therefore it at once gives the essential properties of nourishing the frame and increasing its growth and strength. Where nourishment is necessary for the restoration of certain parts of the body, it is most highly useful. Ten grains given to an adult, combined with sugar, in the form of a powder, or with strong barley-water, will be more beneficial than four grains of the disulphate of quinine, and will even do more than that most useful preparation, under certain circumstances, in the restoration of various parts of the animal economy. After reading the observations of Liebig respecting proteine, I was induced to administer it to several patients, and have carefully watched its effects; and from my personal observation can most strongly recommend it as a medicine likely to prove highly beneficial. In scrofula its exhibition has been extremely serviceable, and no doubt it will prove very useful in other diseases. In a constitution where the milk is not of a sufficient quality to satisfy the child or to give it nourishment, or where the mother feels weak and debilitated from the act of nursing, proteine will be of service, not in the shape of diet, but in a medicinal point of view, to strengthen the secretion and the system generally, not only of the mother but also of the child." P. 28.

Amongst the disorders occurring during lactation, Mr. Tuson speaks of Suppression of the Milk, and he mentions a case where both the mammary glands were so injured by an accident as to be incapable after subsequent pregnancies of secreting milk.

"A woman was admitted into the Middlesex Hospital in the year 1826, who had been knocked down by a horse that had run away with a cart, the wheel passed over her chest, which contused and lacerated both the mammary glands; sloughing took place, the wound healed, and the patient was discharged cured. I have occasionally seen her since the accident. She has borne several children, but was obliged to bring them up by hand, as no secretion took place in either of the breasts, although every means were employed to induce the glands to perform their functions." P. 68.

Mr. Tuson disapproves of the use of warm fomentations or poultices in the treatment of milk abscesses, as their tendency is to derive blood to the affected part, and so promote, instead of repress suppuration. He speaks very favourably of a lotion composed of two drachms and a half of hydrochlorate of ammonia in six ounces of rosemary or common spirit, which is to be frequently applied over the affected gland by means of lint saturated with it and covered over with oil silk. The suggestion was derived from an old surgical work which our author chanced to be reading some years ago. But we suppose that there are few practical men who have not been in the habit of using lotions of the hydrochlorate of ammonia without knowing anything of Justerman's observations.

Mr. Tuson quotes at full length Cruveilhier's description of fibrous tumours of the mammæ, without adding to it, and he transcribes Müller's, Dr. William Budd's, and Dr. Copland's descriptions of the varieties of cancer, without attempting to condense or analyse them—and thus we find a large portion of his book made up of lengthy extracts from various authorities. We do not see Dr. Walshe's name amongst the authors on cancer.

Mr. Tuson has described with some care the different remedies which have been employed for the relief or cure of cancer, and we think this part by far the most useful of the book. Mr. Tuson discredits the notion of cancer being curable by any local means whatever, but he speaks highly of some ingredients which, being applied over an open cancer, establish a clean granulating surface, in place of a foul and unhealthy one. He thinks compression of a cancerous breast, either by bandages, with pads, or by the air-cushion recommended by Dr. Arnott, as ingenious methods to promote absorption, but that the effect of this absorption is to transfer the malignant disease to other structures. Chloride of Zinc is regarded by our author as one of the most useful remedies in the treatment of cancer. It may be given internally, and used as a local application. Mr. Tuson has "frequently healed a large ulcerated surface by its use, not only on one occasion, but several times at different periods upon the same patient." A solution of the chloride of zinc may be applied to large irregular cancerous ulcers by means of a syringe, and with great but temporary benefit.

A plaster made with the following ingredients has been found by Mr. Tuson serviceable in many tumours of the breast:—

"Mercurial ointment, one ounce; gum ammoniacum, six drachms; extract of deadly nightshade, four drachms; prussic acid, one drachm: reduce the gum to a fine powder, and with the extract and a little water form a thick mass, and then add the ointment previously mixed with the acid, and unite them by means of a pestle and mortar. This composition is to be thickly spread upon leather, and a piece cut to the size of the tumour and applied carefully over it." P. 410.

We transcribe Mr. Tuson's account of the chloride of carbon, and its use in cancer and other affections. We believe that Mr. Tuson has used this agent extensively, and his experience, with reference to it, is valuable.

"Chloride of carbon is a thick ætherial oily fluid, dissolving slowly in water. When dropped into a glass of that liquid, it may be observed to descend gradually, floating about like some spirits. It has a strong odour of chlorine, not an unpleasant taste diluted with water, and removes the fetor when applied to ulcerated surfaces.

"The first trial of this preparation, which I introduced as a medicine was as a local application to a cancer of the breast, and its effect was of a sedative character. The patient was relieved from a great deal of the pain which she had previously suffered from. It was then given internally, one drop in water at night. It produced sleep, and gave perfect ease. The dose was increased to two and then to three drops; and the patient, after taking a dose of three drops, slept for twenty-eight hours: when she awoke, she appeared and said she felt as if she had been intoxicated. In this case the remedy produced sloughing of the diseased structure. In very many cases that have come under my treatment, this remedy has produced perfect freedom from pain, quieted the mind and nervous system generally, prevented the rapid growth and progress of the disease; and has rendered the patient's life comparatively happy to their previous feeling and condition. In some cases it has little or no effect; in a few instances affecting the head, and making the symptoms more aggravated. When sleep is not produced by one or two drops of this preparation, a very small dose of the solution of acetate of morphine will produce immediate effect in such cases where it is likely to be serviceable. Acids will also produce the same result. In cases of uterine irritation or neuralgic affections of that organ, it has proved most highly beneficial, not only in allaying the pain, but by producing a perfect cure. In such cases its exhibition has been internally; but in others attended with obstinate discharge, it should be used as an injection as well as being prescribed internally. In violent sickness, when all the usual and most approved remedies have failed to allay the vomiting, three drops of this preparation has at once taken effect; I have in such cases found benefit from applying it locally to the pit of the stomach. In cancer of the pylorus it has proved most efficacious in preventing the return of the food, and in relieving the pain and sufferings of the patient. In sloughing ulcers I have used it extensively, and I am not acquainted with a remedy more beneficial. In phagedæna there cannot be a more useful local application; but care should be taken to apply it only to the sloughing parts. The usual strength as a local application is a drachm of the chloride of carbon to a pint of water; either as a lotion or an injection, it has been frequently prescribed with poppy decoction, and extract of conium, and as an injection in this form its effect has been very successful. The dose internally is from one to four or five drops; but in cases of malignant diseases, one to two or three drops will be quite sufficient to produce sleep, if it is likely to produce this effect. Like all remedies, it does not act upon every constitution alike; but if any person doubts its effects, the better way would be to take a dose of three drops and watch the result: it is a safe remedy. In cases where it has been continued for a length of time (several months), it has been observed to produce a state of debility in the system which can be remediated by the exhibition of the ammoniated tincture of bark, or sesquicarbonate of ammonia. The preparations of steel will also be useful under such circumstances. In fungoid disease, the application of the chloride of carbon has been very remarkable: it was applied to a diseased structure extending from the mastoid process to near the centre of the clavicle: the whole of the tumour sloughed and the part healed. It was afterwards applied to a diseased structure below the outer part of the knee-joint: this tumour sloughed, the swelling was about the size of an orange, and the slough came away from an orifice in the skin the size of a shilling, and this also healed. It was then applied to a tumour in the groin, which also sloughed and healed. Its application was then employed to a swelling over the shoulder; but at this period, the disease made rapid progress over the abdomen, and the patient died. Had she lived, no doubt it might have had the same effect upon the last swelling; when she was admitted into the hospital these tumours were all formed. Since this period I have used this application to a fungoid tumour of the breast. It produced a slough, and the wound afterwards healed; at present the patient has had no return of the disease. I am induced to believe it may prove beneficial in this class of disease.

" Chloride of carbon mixed with water forms a very useful gargle in foul ulcerated sore throats, removing the fetor and giving the ulcers a healthy appearance. It is also of the greatest use in affections of the gums and teeth, removing the unpleasant stinging pains produced by the exposure of some nervous filament; and its use not only gives ease, but removes any unpleasant fetor from the breath. It may be used with a common tooth-brush, instead of any other application to the teeth. In neuralgic affections it has given the greatest ease, by being employed in the form of a liniment, composed of soap liniment, two ounces, to one drachm of chloride of carbon, or with camphorated oil. This liniment should be carefully rubbed over the part affected." P. 416.

Mr. Tuson speaks favourably of the chloride of lead in producing a healthy surface on a cancerous sore, and in relieving the pain in "painful neuralgic tumors."

